# A shotgun proteomic dataset of human mucosal-associated invariant T cells

**DOI:** 10.1016/j.dib.2024.110786

**Published:** 2024-07-31

**Authors:** Harshi Weerakoon, John J. Miles, Michelle M. Hill, Ailin Lepletier

**Affiliations:** a QIMR Berghofer Medical Research Institute, Herston, QLD, Australia; b School of Biomedical Sciences, The University of Queensland, Brisbane, QLD, Australia; c Department of Biochemistry, Faculty of Medicine and Allied Sciences, Rajarata University of Sri Lanka, Saliyapura, Sri Lanka; d Australian Institute of Tropical Health and Medicine, James Cook University, Cairns, QLD, Australia; e Centre for Clinical Research, Faculty of Medicine, The University of Queensland, Brisbane, QLD, Australia; f Institute for Biomedicine and Glycomics, Southport, QLD, Australia

**Keywords:** Shotgun proteomics, MAIT cells, Unconventional T cells, Human CD8^+^ T cells

## Abstract

Mucosal-associated invariant T (MAIT) cells represent a unique unconventional T cell population important in eliciting immunomodulatory responses in a range of diseases, including infectious diseases, autoimmunity and cancer. This innate-like T cell subset predominantly express CD8 in humans. Unlike conventional CD8^+^ T cells, which recognize peptide antigen presented by polymorphic major histocompatibility complex (MHC) molecules, MAIT cells are restricted by MR1, a non-polymorphic antigen-presenting molecule widely expressed in multiple tissues. Thus, identification of proteomic signature of MAIT cells in relation to conventional T cells is pivotal in understanding it's specific functional characteristics. The high-resolution dataset presents here comprehensively describes and compare the whole cell proteomes of MAIT (TCRVα7.2^+^CD161^+^) and conventional/non-MAIT T cells (TCR Vα7.2^−^CD161^−^) in humans. The dataset was generated using the proteomic samples prepared from matched T cell subsets sorted from peripheral blood mononuclear cells (PBMC) of three healthy volunteers. Peptides obtained from trypsin-digested cell lysates were analysed using Data-Dependent Mass Spectrometry (DDA-MS). Label-free quantitation of DDA-MS data using MaxQuant and MaxLFQ software identified 4,442 proteins at a 1 % false discovery rate. Of them, 3680 proteins that were detected with single UniProt accession and a minimum of 2 unique or razor peptides were assessed to identify differentially abundant proteins between MAIT cells and conventional T cells, including total T cells and CD8^+^ T cells. The dataset comprises high-quality label-free quantitative proteomic data that can be used to compare the expression pattern of whole cell proteomes between the above-mentioned T cell populations. Further, this can be used as a reference proteome of human MAIT cells for the in-depth understanding of the MAIT cell behaviour among T cells and to discover potential therapeutic targets to modulate MAIT cell function.

Specifications Table

**Table:** 

Subject	Immunology
Specific subject area	Mucosal-associated invariant T (MAIT) cells are unconventional T cells important in human immunity. However, few studies have examined the primary human MAIT cell proteome. As majority of MAIT cells express CD3^+^ and CD8^+^, this dataset compares the proteome of MAIT cells with matched conventional T cells (total CD3^+^ and CD3^+^CD8^+^ T cells) circulating in the blood of healthy volunteers to establish differentially abundant proteins.
Type of data	Tables, Figures, Raw and Processed data
Data collection	Label-free shotgun data were generated from MAIT, and conventional/non-MAIT T cells purified from the blood of three healthy volunteers. For sorting, CD3^+^, CD161^high^, and TCR Vα7.2^+^ cells were gated as MAIT cells. Peptide samples obtained from trypsin-digested cell lysates were analysed using an Orbitrap Fusion^TM^ Tribrid^TM^ mass spectrometer (Thermo Fisher Scientific, USA) inline coupled to nanoACQUITY ultra-performance liquid chromatography system (Waters, USA). Peptides were separated using a 160-minute chromatographic gradient at 0.3 µl/min flow rate. Raw proteomic data were analysed and normalized using MaxQuant (Release 1.6.0.16) and MaxLFQ software respectively.
Data source location	Raw proteomic data are available via ProteomeXchange [1]. Data were generated from volunteers recruited at QIMR Berghofer Medical Research Institute -Brisbane, Queensland - Australia.
Data accessibility	Repository name: ProteomeXchange via PRIDE database Data identification number: PXD052574 https://www.ebi.ac.uk/pride/archive/projects/PXD052574 - reviewer_pxd052574@ebi.ac.uk - https://www.ebi.ac.uk/pride/review-dataset/3d4ae97c1c1d4edd9dda94c7a9824e23 https://doi.org/10.1016/J.DIB.2024.110786

## 1 Value of the Data

• The dataset generated by label-free shotgun proteomic approach enables the comparison of approximately 3600 proteins between human MAIT and conventional T cells (including total CD3^+^ T cells, and CD3^+^CD8^+^ T cells).
• Researchers can use this dataset to explore the phenotypic and functional characteristics of human MAIT cells and differentiate them from conventional T cells.
• As the dataset was generated from peripheral blood mononuclear cells (PBMC) collected from normal healthy adults, it can be used as an exploratory proteome when characterizing changes in MAIT cell proteome associated with multiple conditions.

## 2 Background

Mucosal-associated invariant T (MAIT) cells are evolutionary conserved, unconventional T cells, characterized by the expression of semi-invariant T cell receptor (TCR) with a canonical TRAV1-2/TRAJ33 (Vα7.2/Jα33) that can recognize vitamin B metabolites derived from some bacteria and fungi [2]. Their immunomodulatory functions are mainly associated with secretion of cytotoxic molecules [3] and cytokines [[4], [5], [6]]. In humans, MAIT cells are found in mucosal tissues [7,8], peripheral blood [9] and liver [10,11]. MAIT cells represent approximately 10 % of circulating T cells and present a memory phenotype that allow them to rapidly respond to stimulus in a range of pathological conditions [10,12]. Since their discovery 15 years ago, omics analysis of human MAIT cells have evidenced their phenotypic and functional characteristics [[13], [14], [15]]. MAIT cells are classified under the common T cell antigen, CD3, and primarily express CD8 in humans, which is a canonical marker for conventional cytotoxic T cells [7]. Therefore, describing the proteomic demarcation of MAIT cells in relation to conventional T cell populations is crucial for identifying their unique functional and phenotypic properties.

## 3 Data Description

**Fig. 1:**
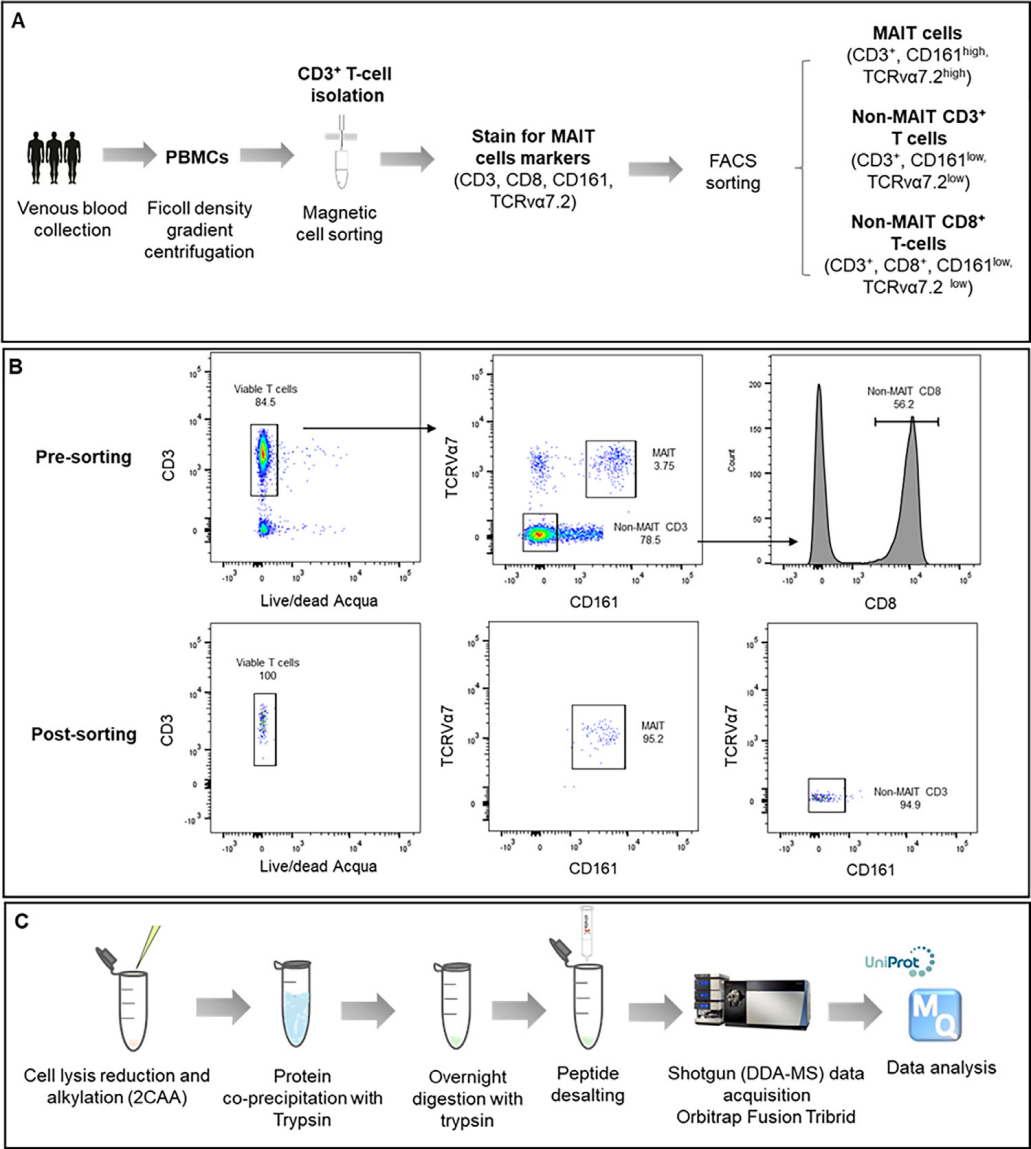
Experimental design used to generate the proteomic data. **A**. Key steps for isolating T cell populations **B.** Gating strategy used for fluorescence-activated cell sorting (FACS) to obtain MAIT cells and non-MAIT T cell populations at a high purity**.** Gating strategy is shown for cells pre and post-FACS. **C.** Key steps followed for the obtention of trypsin digested peptide samples and proteomic data acquisition.

The dataset presented in this article includes label-free quantitative proteomic data for human MAIT (CD3^+^TCR Vα7.2^+^CD161^+^) and conventional T cells (including total CD3^+^ and CD8^+^ T cells bearing a TCR Vα 7.2^−^CD161^−^ phenotype). The data were generated using a Data-Dependent Acquisition approach (DDA-MS) with a Orbitrap Fusion^TM^ Tribrid^TM^ mass spectrometer (Thermo Fisher Scientific, USA) inline coupled to nanoACQUITY ultra performance liquid chromatographic (Waters, USA) system. The method used to isolate the cell populations, as well as the key steps for proteomic sample preparation and data acquisition, are summarized in Fig. 1. Raw data were analyzed using MaxQuant software (Release 1.6.0.16) [16], with the analysis conducted against the UniProt human-reviewed proteome. MaxLFQ was employed to normalize the protein expression data for label-free quantification [17]. All raw and processed data are deposited and publicly available through the ProteomeXchange data repository (PXD052574), as summarized in Table 1.

**Table 1:** Data files available through the ProteomeXchange data repository (PXD052574).

	File/folder	Description
1	Rep1_MAIT.raw	.raw file of MAIT cells – Biological Replicate 1
2	Rep2_MAIT.raw	.raw file of MAIT cells – Biological Replicate 2
3	Rep3_MAIT.raw	.raw file of MAIT cells – Biological Replicate 3
4	Rep1_nonMAIT_CD3.raw	.raw file of nonMAIT CD3^+^ T cells – Biological Replicate 1
5	Rep2_nonMAIT_CD3.raw	.raw file of nonMAIT CD3^+^ T cells - Biological Replicate 2
6	Rep3_nonMAIT_CD3.raw	.raw file of nonMAIT CD3^+^ T cells – Biological Replicate 3
7	Rep1_nonMAIT_CD8.raw	.raw file of nonMAIT CD8^+^ T cells – Biological Replicate 1
8	Rep2_nonMAIT_CD8.raw	.raw file of nonMAIT CD8^+^ T cells - Biological Replicate 2
9	Rep3_nonMAIT_CD8.raw	.raw file of nonMAIT CD8^+^ T cells – Biological Replicate 3
10	search.zip	MaxQuant ouput files resulted from the analysis of the above .raw files against UniProt/SwissProt human reviewed proteome
11	parameters.txt	Parameters used in the data analysis through MaxQuant, MaxLFQ search engine
12	human_proteome_reviewed_25102017.fasta	UniProt/SwissProt proteome database used in the analysis
13	MaxQuant_MaxLFQ_Output_protein group file.txt	MaxQuant ouput files giving the protein quantification data and LFQ normalised protein intensities

**Fig. 2:**
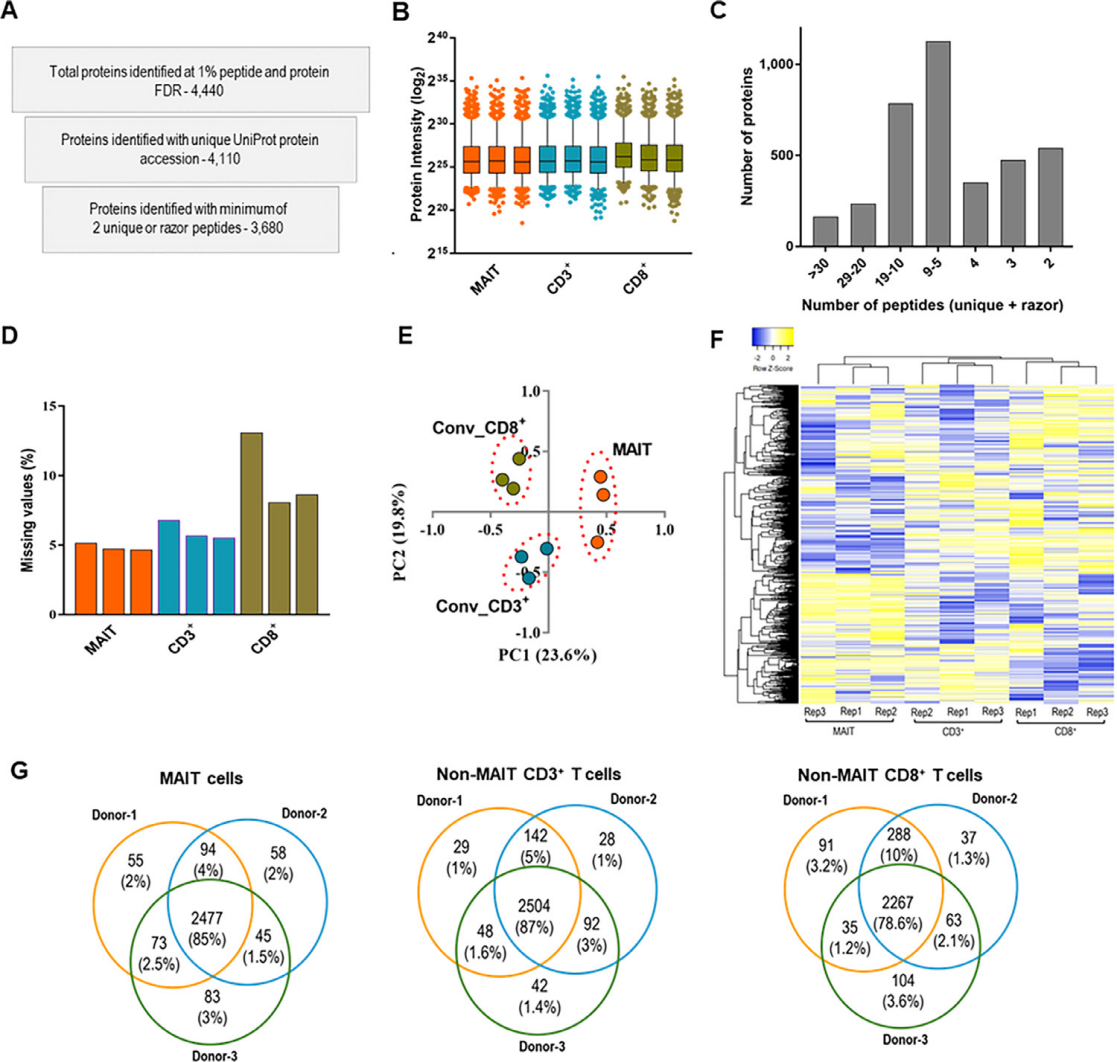
An overview of the proteomic dataset obtained from MAIT and conventional/non-MAIT T cells. **A**. Number of proteins obtained at various stages of data curation **B.** Distribution of normalized protein intensities across different samples (central lines and boxes represent means and 95 % confidence intervals respectively while whiskers are 2.5 to 97.5 percentiles) **C.** Number of proteins identified and quantified with varying number of peptides **D.** Percentage of proteins with missing values in each cell population (percentage was obtained from the total number of identified proteins) **E.** Principal component analysis of protein intensity data **F.** Heat map showing the hierarchical clustering of quantified protein intensity data. **G.** Venn diagrams ilustrating the variations in protein expression across three donors for all cell populations. Within each T cell group, these diagrams visualize both common and differential proteins across the donors.

The parameter file deposited with the dataset guides the researchers on the criteria used in the identification and quantification of peptides and proteins. The current analysis has led to the detection and quantification of 4440 protein groups at a peptide and protein false discovery rate (FDR) of 1 %. In future applications, the raw data can be reanalyzed with different parameters depending on the study objectives. Of the identified protein groups in the current analysis, 4110 proteins (93 % of total) had a single UniProt accession name and 3680 proteins (83 % of total) were detected with a minimum of 2 unique or razor peptides (Fig. 2A). To assess the quality of the selected proteins, the data were further analyzed to determine the normalized protein intensity distribution (Fig. 2B), the number of peptide ions detected per protein (Fig. 2C), and the percentage of missing protein intensity values in each sample (Fig. 2D). The results of the principal component analysis and unsupervised hierarchical clustering of proteomic data from the different T cell subsets are shown in Fig. 2E and 2F, respectively. The protein expression variation (fold change) across different donors is presented in Fig. 2G.

**Fig. 3:**
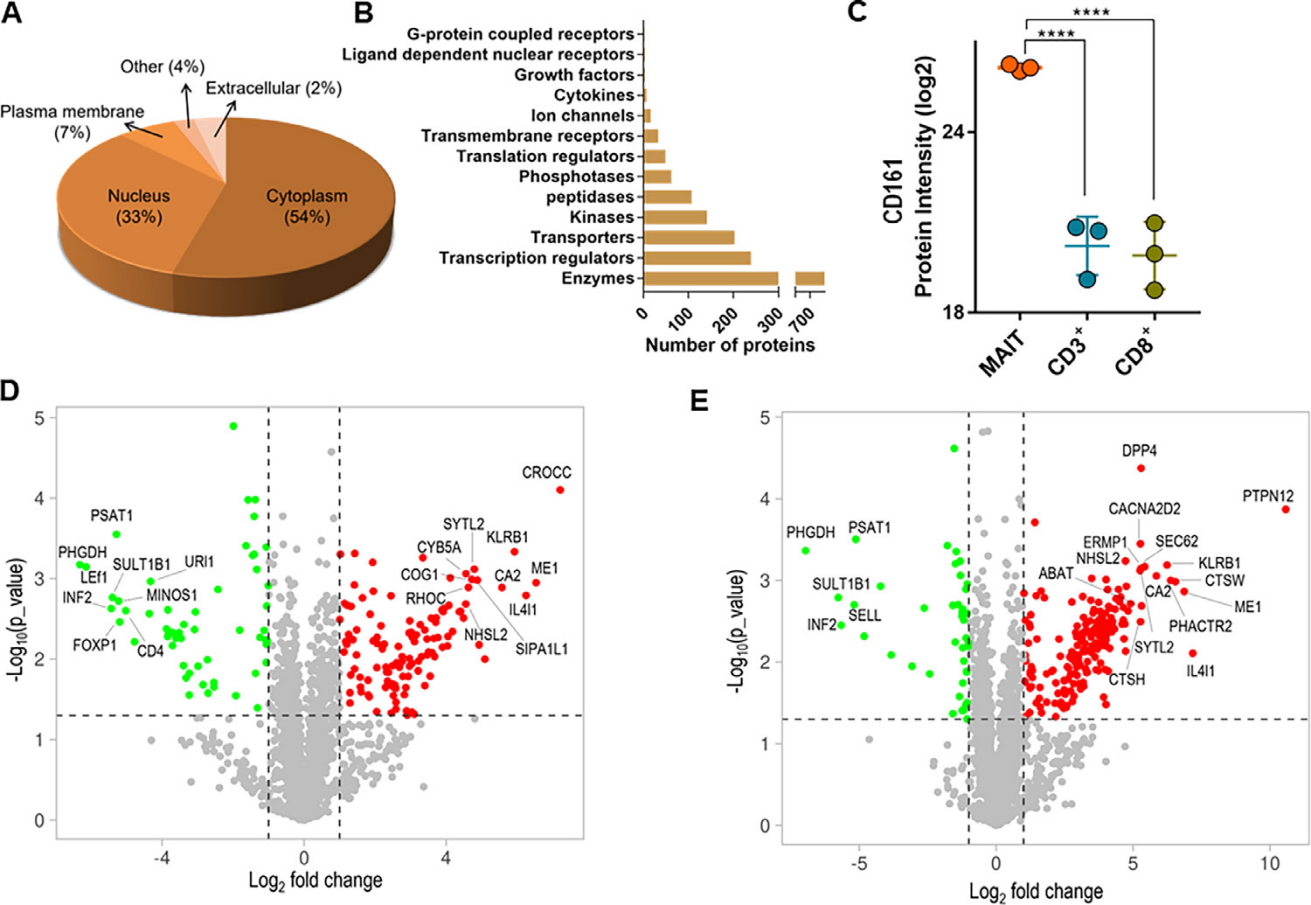
Gene ontology and differential expression analysis of normalized protein intensity data. **A**. Distribution of proteins in different subcellular compartments is given as a percentage of all proteins selected for differential expression analysis. **B.** Distribution of proteins selected for differential expression analysis across different functional groups (Qiagen, IPA). **C.** Expression of CD161 in three cell subsets as based on DDA-MS data (****q < 0.0001, multiple t-test with false discovery determination by two-stage linear step-up procedure of Benjamini, Krieger and Yekutieli) **D**. Volcano plots labelling the top 20 differentially expressed proteins in MAIT cells compared to non-MAIT CD3^+^ T cells **E.** Volcano plots labelling the top 20 differentially expressed proteins in MAIT cells compared to non-MAIT CD8^+^ T cells.

**Fig. 4:**
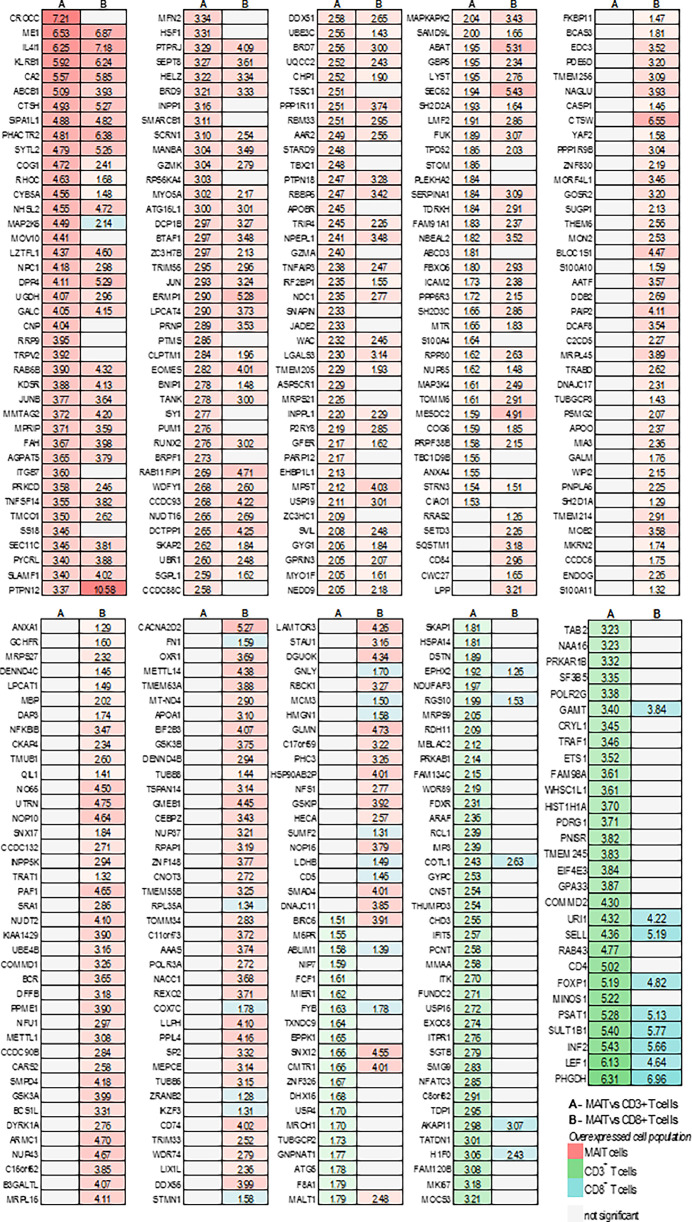
Differentially expressed proteins in MAIT cells compared to CD3^+^ and CD8^+^ conventional/non-MAIT T cell populations. Proteins with ±log_2_fc≥1 and a q value of 0.05 were considered differentially expressed.

The current analysis excluded the proteins with missing expression data in more than 50 % of samples and those with m-score of below 5 when calculating differential expression across the three T cell populations. As per the subcellular (Fig. 3A) and functional group (Fig. 3B) analysis, about half (54 %) of the selected proteins (*n =* 1566) were mainly present in the cytoplasm and 28 % were classified as enzymes (*n =* 798). As expected, differential expression analysis revealed significant overexpression of CD161 in MAIT cells (Fig. 3C). In total, 243 (∼8 %) and 285 (∼10 %) proteins were differentially expressed (DE) in MAIT cells compared to conventional CD3^+^ and CD8^+^ T cells, respectively. The top 20 DE proteins in MAIT cells compared to CD3^+^ and CD8^+^ are shown in Fig. 3D and 3E. These figures demonstrate that the majority of DE proteins are overexpressed in MAIT cells (Fig. 3D and 3E). Further, the DE proteins and canonical pathways are summarized in Fig. 4 and Table 2, respectively.

**Table 2:** Canonical pathways enriched by differentially expressed proteins in MAIT cells compared to Non-MAIT T cells.

	Ingenuity Canonical Pathways	Log_2_ fold-change	Over-expressed	Under-expressed	Quantified proteins
MAIT cells vs non-MAIT CD3^+^ T cells	B cell receptor signalling	1.4	9/192 (5 %)	1/192 (1 %)	MAP2K6, RRAS2, JUN, INPPL1, GSK3A, MAP3K4, INPP5K, GSK3B, MALT1, NFKBIB
	14-3-3-mediated signalling	1.4	8/131 (6 %)	0/131 (0 %)	RRAS2, JUN, TUBB6, TUBB8, PRKCD, EDC3, GSK3A, GSK3B
	CD40 signalling	1.4	5/79 (6 %)	1/79 (1 %)	MAP2K6, TANK, JUN, TNFAIP3, MAPKAPK2, NFKBIB
	TNFR2 signalling	1.4	4/30 (13 %)	0/30 (0 %)	TANK, JUN, TNFAIP3, NFKBIB
	Regulation of IL-2 in activated and anergic T-cells	1.4	6/80 (8 %)	0/80 (0 %)	RRAS2, JUN, CHP1, SMAD4, MALT1, NFKBIB
	Protein kinase A signalling	1.39	11/401 (3 %)	3/401 (1 %)	CHP1, PTPN18, PPP1R11, GSK3A, PTPN12, AKAP11, PTPRJ, PRKCD, SMAD4, LEF1, H1F0, GSK3B, NFKBIB, PDE6D
	TNRF2-mediated oxidative stress response	1.38	8/193 (4 %)	1/193 (1 %)	MAP2K6, DNAJC17, RRAS2, JUN, PRKCD, JUNB, GSK3B, SQSTM1, DNAJC11
	3-phosphoinositide degradation	1.38	7/157 (4 %)	1/157 (1 %)	PTPRJ, PIP4P1, NUDT16, EPHX2, INPPL1, INPP5K, NUDT2, PTPN12
MAIT cells vs non-MAIT CD8^+^ T cells	CD40 signalling	1.44	5/79 (6 %)	1/79 (1 %)	MAP2K6, TANK, JUN, TNFAIP3, MAPKAPK2, TRAF1
	TNFR2 signalling	1.44	3/30 (10 %)	1/30 (3 %)	TANK, JUN, TNFAIP3, TRAF1
	Thiosulfate disproportionation III (Rhodanese)	1.44	2/3 (66 %)	0/3 (0 %)	MPST, MOCS3
	Protein kinase A signalling	1.44	6/401 (1 %)	7/401 (2 %)	NFATC3, CHP1, PTPN18, PPP1R11, ITPR1, PTPN12, AKAP11, HIST1H1A, PTPRJ, PRKCD, PRKAR1B, LEF1, H1F0
	CD28 signalling in Th cells	1.42	2/132 (2 %)	5/132 (4 %)	JUN, NFATC3, CD4, CHP1, ITPR1, MALT1, ITK

## 4 Experimental Design, Materials and Methods

### 4.1 Purification of primary human MAIT, CD3^+^, and CD8^+^ T cell populations

Human circulating MAIT cells were isolated from peripheral blood mononuclear cells (PBMCs) obtained from three healthy young volunteers aged between 30-35 years (2 males and 1 female). To isolate MAIT cells, first CD3^+^ T cells were negatively enriched from PBMCs using a pan-human T cell isolation kit (Miltenyi Biotec, USA) and magnetic activated cell sorting. CD3^+^ T cells were surface stained with live/dead Fixable Aqua (Life Technologies, USA), CD3-APCe780 (clone SK7; eBioscience, Thermo Fisher Scientific, USA), CD161-APC (clone HP-3G10; eBioscience, Thermo Fisher Scientific, USA), TCR Vα7.2-FITC (clone 3C10; Biolegend, USA) and CD8-Percp/cy5.5 (clone SK1, Biolegend, USA) by incubating the cells for 20 minutes at 4°C in dark. After washing three times with cold FACS buffer the stained cells were sorted using a FACS Aria III flow cytometer (BD bioscience, USA) to obtain ∼ 1×10^6^ CD3^+^, CD8^+^, and MAIT cells from each donor. In the FACS sorting, CD3^+^, CD161^+^, and TCR Vα7.2^+^ cells were sorted as MAIT cells while CD3^+^, CD161^−^ and TCR Vα7.2^−^ and CD3^+^, CD8^+^, CD161^−^ and TCR Vα7.2^−^ were collected as CD3^+^ and CD8^+^ conventional T cells, respectively (Fig. 1A and B). Collected cells were washed three times with cold PBS, pelleted, and stored at – 80°C for proteomic sample preparation.

### 4.2 Proteomic sample preparation

The steps used in the proteomic sample preparation and data acquisition are shown in Fig. 1C. The cell pellets were thawed and lysed in a lysis buffer composed of 2 % sodium dodecyl sulphate (Biorad, USA) in 100 mM triethylammonium bicarbonate (TEAB, Sigma-Aldrich, USA) and 1 x Roche complete protease inhibitor cocktail (Sigma-Aldrich, USA). Then 200 ng of ovalbumin (Sigma-Aldrich, USA) was added as an internal standard. The amount of protein in each cell lysate was quantified at a wavelength of 562 nm using Pierce bicinchoninic acid (BCA) protein assay (Thermo Fisher Scientific, USA), following the manufacturer's instructions. About 20 µg of protein from each cell lysate was reduced in 10 mM of tris (2-carboxyethyl) phosphine hydrochloride (Thermo Fisher Scientific, USA) at 60°C for 30 minutes and alkylated in 40 mM chloroacetamide (Sigma, USA) at 37°C in dark for 45 minutes. Cell lysates were then co-precipitated overnight with sequencing grade modified porcine trypsin (Promega, USA) at a trypsin: protein ratio of 1:100 in cold (-20°C) cold chromAR grade methanol (Honeywell Research Chemicals, USA) as described previously [18]. On the next day, samples with precipitated proteins were further cleaned by washing the pellet three times consecutively with 100 %, 90 %, and 100 %, cold chromAR grade methanol (Honeywell Research Chemicals, USA). Each centrifugation was performed for 15 min at 16,100xg at 4°C and the supernatants were aspirated carefully without disturbing the protein pellets. Resulted protein pellets were then resolubilized in 50 mM TEAB containing 5 % acetonitrile (ACN, Honeywell research chemicals, USA) and were incubated in a thermo-mixture at 37°C for 2 h at 600 rpm after adding 1 µl of 1 µg/µl sequencing grade trypsin. At the end of the incubation period, another 1 µl of 1 µg/µl (1:50) trypsin was added, vortexed to mix, and incubated overnight to obtain complete protein digestion. After 12 hours of digestion, the enzymatic reaction was inhibited by adding 25 µl of 5 % formic acid (Sigma-Aldrich, USA), and the resulting acidified tryptic digested peptides were desalted using strata-x polymeric reversed phase 10 mg/ml C18 cartridges (Phenomenex, USA). Desalted peptides were dried using a speedVac vacuum concentrator (Thermo Fisher Scientific, USA) at 35°C and stored at -80°C until tandem mass spectrometry (LC-MS/MS) based proteomic analysis.

### 4.3 DDA-MS data acquisition

LC-MS/MS analysis of desalted peptide samples was performed on an Orbitrap Fusion^TM^ Tribrid^TM^ mass spectrometer (Thermo Fisher Scientific, USA) inline coupled to nanoACQUITY ultra performance liquid chromatographic (Waters, USA) system. From each sample, ∼ 1 µg of peptides as quantified by micro-BCA (Thermo Fisher Scientific, USA) was loaded onto a Symmetry C18, 2G, VM (100Å, 5 µm particle size, 180 µm x 20 mm) trap column (Waters, USA) at a flow rate of 0.3 µL/min to separate the peptides on a BEH C18 (130Å, 1.7 µm particle size, 75 µm x 200 mm) column (Waters, USA). The mobile phase consisted of buffer A (0.1 % formic acid), and buffer B (100 % acetonitrile and 0.1 % formic acid) was used to create three consecutive linear gradients (buffer B, 5 %- 9 % between 3 and 10 min, 9 %-26 % between 10 and 120 min and 26 %-40 % between 120 and 145 min) to elute the peptides. After elution, the column was washed with buffer B at a concentration of 40 %- 80 % between 145 and 152 min, then holding it at 80 % until 157 min and at 1 % until 160 min. The eluted peptides were ionized using Nanospray Flex ion source (Thermo Fisher Scientific, USA) in which the ion spray voltage and heating temperature were held at 1.9 kV and 285°C respectively. In DDA-MS acquisition, Chromeleon software (version 6.8, Dionex) included in Xcalibur software (version 3.0.63, Thermo Fisher Scientific, USA) was used to control the liquid chromatographic system. Peptide ions in the mass range of 380 – 1500 m/z were selected at 120,000 FWHM resolution to generate MS1 spectra. The mass spectrometer was controlled by Xcalibur software to operate “top speed” mode allowing automatic selection of positively charged (+2 to +7) top 15 peptides to trigger MS2. Higher Energy C-trap Dissociation (HCD) was used to fragment the selected peptide ions. In the acquisition of MS2 spectra, the resolution and dynamic exclusion time were set as 30,000 FWHM and 90 seconds respectively. The cycle time was 2 s.

#### 4.3.1 Data processing and statistical analysis

MaxQuant (Release 1.6.0.16) software [16] was used to process the .raw files in which spectral data were searched against UniProt human-reviewed proteome database containing 20,242 entries (downloaded on 25^th^ October 2017). MaxLFQ included in MaxQuant software was used to obtain the normalized label-free peptide and protein intensity data [17]. Trypsin-digested peptides with a maximum of 2 miscleavages were included in the analysis. Only carbamidomethylation of cystine (fixed modification), and oxidation of methionine and N terminal acetylation (variable modifications) peptide modifications were allowed. Precursor and product mass tolerance were set as ± 20 ppm and ± 40 ppm respectively to identify the peptides up to the maximum charge of +7. Only the peptide spectral matches and proteins detected at a 1 % of FDR were selected in which the proteins that were detected with at least one unique or razor peptide were quantified between runs.

In the downstream analysis, less reproducible proteins (expression data is missing for > 50 % of samples) and that were quantified at m-score of < 5 were removed from the final quantification and the missing values of the remaining proteins were imputed using maximum likelihood estimate (R package) [19]. In the statistical analysis, mean intensity values of each cell population were compared using multiple t-test with FDR determination by two-stage linear step-up procedure of Benjamini, Krieger and Yekutieli [20] to identify the differentially abundant proteins in MAIT cells compared to conventional CD3^+^ and CD8^+^ Tcells. Expression fold change was obtained in the log_2_ fold change (log_2_fc) scale to depict proteins expressed at ±log_2_fc≥1 at q value < 0.05 as differentially abundant proteins. Qiagen ingenuity pathway analysis (QIAGEN Inc., https://digitalinsights.qiagen.com/IPA) was used to profile the subcellular localization, biological functions of the selected proteins, and the canonical pathways enriched by the differentially abundant proteins [21]. In IPA analysis, the *p*-value corrected for false discoveries in multiple comparisons using Benjamini-Hochberg (B-H p value) equation was used to set the cut-off, in which canonical pathways identified at or above 1.30 –log_10_ B-H p value (B-H *p* value = 0.05) were considered significant.

## Limitations

The present analysis aimed to characterize the proteome of human circulating MAIT cells in three young healthy volunteers. The proteome of MAIT cells can vary, particularly in elderly people, children, and those with different disease conditions and this diversity is not represented in the current dataset. As non-MAIT T cells were sorted from CD3^+^ T cells, a contamination with non-MAIT unconventional T cell populations (e.g. Vδ2 γδ T cells), which have some similarities to MAIT cells can be expected. Further, CD3^+^ and CD8^+^ conventional T cell populations contain T cells of different phenotypes (eg; naïve and memory) while MAIT cells predominantly have memory phenotype. Thus, some differentially expressed proteins will not relate to differences between MAIT and conventional T cells, but rather to differences in the ratio of naïve vs. memory cells. As indexed retention time (iRT) peptides (Biognosys AG, Switzerland) were not added to the samples during DDA-MS data acquisition, the use of this data to develop spectral libraries for data independent analysis (DIA) will be limited.

## Ethics Statement

Ethical clearance for this study was obtained from the QIMRB human research ethics committee (HREC, #P2058). Informed consent was obtained from all volunteers and the study adhered to the Declaration of Helsinki of 1975.

## CRediT Author Statement

**Harshi Weerakoon:** Data curation; Formal analysis; Validation; Investigation; Methodology; Writing - original draft. **John J Miles:** Conceptualization; Methodology; Resources; Supervision; Project administration; Funding acquisition, **Michelle M Hill:** Conceptualization; Methodology; Resources; Supervision; Writing-original draft; Project administration. **Ailin Lepletier:** Conceptualization; Methodology; Supervision; Writing-Review & Editing; Project administration.

## Data Availability

Human mucosal-associated invariant T (MAIT) cell proteome (Original data) (ProteomeXchange via the PRIDE database)

## References

[1] Perez-Riverol Y., Csordas A., Bai J., Bernal-Llinares M., Hewapathirana S., Kundu D.J., Inuganti A., Griss J., Mayer G., Eisenacher M., Pérez E., Uszkoreit J., Pfeuffer J., Sachsenberg T., Yilmaz S., Tiwary S., Cox J., Audain E., Walzer M., Jarnuczak A.F., Ternent T., Brazma A., Vizcaíno J.A. (2019). The PRIDE database and related tools and resources in 2019: improving support for quantification data. Nucleic Acids Res..

[2] Godfrey D.I., Koay H.F., McCluskey J., Gherardin N.A. (2019). The biology and functional importance of MAIT cells. Nat. Immunol..

[3] Kurioka A., Ussher J.E., Cosgrove C., Clough C., Fergusson J.R., Smith K., Kang Y.-H., Walker L.J., Hansen T.H., Willberg C.B., Klenerman P. (2015). MAIT cells are licensed through granzyme exchange to kill bacterially sensitized targets. Mucosal Immunol.

[4] Tomura M., Maruo S., Mu J., Zhou X.Y., Ahn H.J., Hamaoka T., Okamura H., Nakanishi K., Clark S., Kurimoto M., Fujiwara H. (1998). Differential capacities of CD4+, CD8+, and CD4-CD8- T cell subsets to express IL-18 receptor and produce IFN-gamma in response to IL-18. J. Immunol..

[5] Dusseaux M., Martin E., Serriari N., Péguillet I., Premel V., Louis D., Milder M., Bourhis L.Le, Soudais C., Treiner E., Lantz O. (2011). Human MAIT cells are xenobiotic-resistant, tissue-targeted, CD161hi IL-17-secreting T cells. Blood.

[6] Kang S.-J., Jin H.-M., Won E.J., Cho Y.-N., Jung H.-J., Kwon Y.-S., Kee H.J., Ju J.K., Kim J.-C., Kim U.J., Jang H.-C., Jung S.-I., Kee S.-J., Park Y.-W. (2016). Activation, impaired tumor necrosis factor-α production, and deficiency of circulating mucosal-associated invariant T cells in patients with scrub typhus. PLoS Negl. Trop. Dis..

[7] Martin E., Treiner E., Duban L., Guerri L., Laude H., Toly C., Premel V., Devys A., Moura I.C., Tilloy F., Cherif S., Vera G., Latour S., Soudais C., Lantz O. (2009). Stepwise development of MAIT cells in mouse and human. PLoS Biol..

[8] Gold M.C., Napier R.J., Lewinsohn D.M. (2015). MR1-restricted mucosal associated invariant T (MAIT) cells in the immune response to Mycobacterium tuberculosis. Immunol. Rev..

[9] Lepletier A., Lutzky V.P., Mittal D., Stannard K., Watkins T.S., Ratnatunga C.N., Smith C., McGuire H.M., Kemp R.A., Mukhopadhyay P., Waddell N., Smyth M.J., Dougall W.C. (2019). The immune checkpoint CD96 defines a distinct lymphocyte phenotype and is highly expressed on tumor-infiltrating T cells. Immunol. Cell Biol..

[10] Lee O.-J., Cho Y.-N., Kee S.-J., Kim M.-J., Jin H.-M., Lee S.-J., Park K.-J., Kim T.-J., Lee S.-S., Kwon Y.-S., Kim N., Shin M.-G., Shin J.-H., Suh S.-P., Ryang D.-W., Park Y.-W. (2014). Circulating mucosal-associated invariant T cell levels and their cytokine levels in healthy adults. Exp. Gerontol..

[11] Kurioka A., Walker L.J., Klenerman P., Willberg C.B. (2016). MAIT cells: new guardians of the liver. Clin. Transl. Immunol..

[12] Magalhaes I., Solders M., Kaipe H., Kaipe H., Magalhaes I. (2020). MAIT Cells. Methods Mol. Biol. (Clifton, N.J.).

[13] Fergusson J.R., Smith K.E., Fleming V.M., Rajoriya N., Newell E.W., Simmons R., Marchi E., Björkander S., Kang Y.H., Swadling L., Kurioka A., Sahgal N., Lockstone H., Baban D., Freeman G.J., Sverremark-Ekström E., Davis M.M., Davenport M.P., Venturi V., Ussher J.E., Willberg C.B., Klenerman P. (2014). CD161 defines a transcriptional and functional phenotype across distinct human T cell lineages. Cell Rep..

[14] Dias J., Leeansyah E., Sandberg J.K. (2017). Multiple layers of heterogeneity and subset diversity in human MAIT cell responses to distinct microorganisms and to innate cytokines. Proc. Natl. Acad. Sci. U. S. A..

[15] Bulitta B., Zuschratter W., Bernal I., Bruder D., Klawonn F., von Bergen M., Garritsen H.S.P., Jänsch L. (2018). Proteomic definition of human mucosal-associated invariant T cells determines their unique molecular effector phenotype. Eur. J. Immunol..

[16] Cox J., Mann M. (2008). MaxQuant enables high peptide identification rates, individualized p.p.b.-range mass accuracies and proteome-wide protein quantification. Nat. Biotechnol..

[17] Cox J., Hein M.Y., Luber C.A., Paron I., Nagaraj N., Mann M. (2014). Accurate proteome-wide label-free quantification by delayed normalization and maximal peptide ratio extraction, termed MaxLFQ. Mol. Cell. Proteomics..

[18] Weerakoon H., Potriquet J., Shah A.K., Reed S., Jayakody B., Kapil C., Midha M.K., Moritz R.L., Lepletier A., Mulvenna J., Miles J.J., Hill M.M. (2020). A primary human T-cell spectral library to facilitate large scale quantitative T-cell proteomics. Sci. Data..

[19] Team RStudio (2020). http://www.rstudio.com/.

[20] Benjamini Y., Krieger A.M., Yekutieli D. (2006). Adaptive linear step-up procedures that control the false discovery rate. Biometrika.

[21] Krämer A., Green J., Pollard J., Tugendreich S. (2014). Causal analysis approaches in ingenuity pathway analysis. Bioinformatics.

